# Protective Effects of a Polyphenol-Rich Extract from *Syzygium cumini* (L.) Skeels Leaf on Oxidative Stress-Induced Diabetic Rats

**DOI:** 10.1155/2018/5386079

**Published:** 2018-06-26

**Authors:** Vinicyus Teles Chagas, Rafaella Moraes Rego de Sousa Coelho, Renato Simões Gaspar, Samira Abdalla da Silva, Mauricio Mastrogiovanni, Cáritas de Jesus Mendonça, Maria Nilce de Souza Ribeiro, Antonio Marcus de Andrade Paes, Andres Trostchansky

**Affiliations:** ^1^Department of Physiological Sciences, Federal University of Maranhão, 65080805 São Luís, MA, Brazil; ^2^Department of Biochemistry and Center for Free Radical and Biomedical Research, Faculty of Medicine, University de la República, 11800 Montevideo, Uruguay; ^3^Department of Chemistry, Federal University of Maranhão, 65080805 São Luís, MA, Brazil; ^4^Department of Pharmacy, Federal University of Maranhão, 65080805 São Luís, MA, Brazil

## Abstract

*Syzygium cumini* (L.) Skeels has been reported to exert anti-inflammatory and cardiometabolic activities due to its high content of polyphenols. We characterized the chemical composition and assessed the antidiabetic effects of a novel polyphenol-rich extract (PESc) obtained from *S. cumini* leaf. Rats were injected with alloxan (150 mg/kg, ip, ALX group) and followed up for 7 days. Some were orally treated with PESc (50 mg/kg/day) for 7 days before and after diabetes induction (ALX-PP) or only for 7 days after alloxan injection (ALX-P). ALX-P and ALX-PP decreased fasting glycemia in 37 and 43%, respectively, as compared to ALX. Triglycerides and total cholesterol serum levels were also significantly reduced in comparison to ALX. PESc presented high polyphenol concentration (71.78 ± 8.57 GAE/100 g), with flavonoid content of 8.21 ± 0.42 QE/100 g. Upon HPLC-MS/MS and MS/MS studies, five main polyphenols—gallic acid, quercetin, myricetin, and its derivatives—were identified. Myricetin was predominant (192.70 ± 16.50 *μ*g/mg PESc), followed by measurable amounts of gallic acid (11.15 ± 0.90 *μ*g/mg PESc) and quercetin (4.72 ± 0.06 *μ*g/mg PESc). Kinetic assessment of total antioxidant capacity revealed PESc high potency, since maximum response was reached within 5 min reaction time in a concentration-dependent manner. Specific antioxidant activity of PESc was assessed against both DPPH^•^ and ABTS^•+^, showing strong activity (IC_50_: 3.88 ± 1.09 and 5.98 ± 1.19 *μ*g/mL, resp.). PESc also inhibited lipoxygenase activity (IC_50_: 27.63 ± 8.47), confirming its antioxidant activity also on biologically relevant radicals. Finally, PESc induced insulin secretion by directly stimulating INS-1E *β* cells in the absence of any cytotoxic effect. Overall, our results support that PESc is a potent antioxidant phytocomplex with potential pharmacological use as a preventive antidiabetic natural product.

## 1. Introduction

Hyperglycemia induces oxidative stress by producing mitochondrial dysfunction and stress of the endoplasmic reticulum. Oxidative stress, in turn, is involved in the development and progression of type 1 diabetes [1]. Reactive oxygen species cause endothelial lesions that are of fundamental importance for macro- and microvesicular complications of diabetes. In addition, oxidative stress also causes damage to pancreatic *β* cells by activating the autoimmune response in genetically susceptible individuals [2]. Exogenous antioxidants obtained from medicinal plants can neutralize such reactive species and, consequently, their deleterious effects evidenced in diabetes [3].

*Syzygium cumini* (L.) Skeels (*S. cumini*) is a tree from the Indian subcontinent [4] that was brought to Western countries in the mid-nineteenth century, where it started to be used as antidiabetic agent even before the discovery of insulin [5]. This plant species is denominated with a variety of names, *Syzygium jambolanum* (Lam.) DC, *Eugenia jambolana* Lam., *Eugenia cumini* (L.) Druce, and *Myrtus cumini* L., and widely spread in several countries, including Brazil. It is popularly known as jambolão, jambolan, java plum, black plum, Indian blackberry, jaman, jambu, and jambul [6].

Polyphenols are phytochemicals that have been reported to exert healthy benefits [7]. Epidemiological studies show that consuming polyphenol-rich diets is inversely associated with the development of cardiometabolic and neurodegenerative diseases, as well as cancer [8]. *S. cumini* is phytochemically composed of compounds such as hydrolysable tannins, flavonoids, anthocyanins, terpenes, and aliphatic acids [6]. Different parts of *S. cumini* have different compositions but all share a high content of polyphenols [9]. Both fruit and flowers are enriched in anthocyanins as cyanidin, delphinidin, peonidin, pelargonidin, petunidin, and malvidin [10]. The seed contains rutin and quercetin, and leaves have important secondary metabolites such as kaempferol, quercetin, myricetin, and their glycosides [3].

Many important biological activities have been described for *S. cumini* such as hypoglycemic, hypolipidemic, anti-inflammatory, cardioprotective, antibacterial, hepatoprotective, antineoplastic, and antiallergic activities [6, 9]. In fact, a significant antihyperglycemic activity in patients administered with *S. cumini* leaf extract has been previously reported, with this effect being ascribed to the inhibition of the adenosine deaminase protein [11]. Our group reported that *S. cumini* has a variety of polyphenols, including flavonoids, phenolic acids, and tannins, widely distributed in different parts of the plant being potentially protective against cardiometabolic diseases [3]. More recently, we showed that the hydroethanolic extract of *S. cumini* leaf improved the metabolic profile of monosodium L-glutamate- (MSG-) induced obese rats, especially by reverting triglyceride accumulation in both liver and serum [12].

Evidence in the literature suggests that the antihyperglycemic activity of *S. cumini* can be attributed to the antioxidant properties of flavonoids such as quercetin and rutin, which have been identified in *S. cumini* leaf [9]. Thus, in the present report, we took advantage of alloxan-induced diabetic rats, a well-known oxidative stress animal model [13], to characterize the antidiabetic effects of a novel polyphenol-rich extract from *S. cumini* leaf (PESc). Furthermore, main compounds within the enriched extract were identified and chemically characterized as an attempt to correlate them with PESc antioxidant activity *in vitro* and its insulinagogue actions.

## 2. Materials and Methods

### 2.1. Chemical Reagents

All reagents and solvents were of analytical grade. The DPPH^•^ (2,2-diphenyl-1-picryl-hydrazyl-1), ABTS^•+^ (2,2′-azinobis-(3-ethylbenzthiazoline sulfonic acid-6)), quercetin, gallic acid, potassium ferricyanide, ferric chloride, sodium dodecyl sulfate, and hydrochloric acid were obtained from Sigma Chemical Co. (St. Louis, MO, USA). Formic acid and acetonitrile were purchased from Merck (Darmstadt, Germany). Any other solvent used was of HPLC grade.

### 2.2. Botanical Material

Leaves from *S. cumini* plants were collected during September 2012 from specimens located at the campus of the Federal University of Maranhão (São Luís, Maranhão, Brazil). An exsiccated sample of the plant species was identified and stored at the Herbarium of Maranhão (MAR) under the register number 4574.

### 2.3. Sample Preparation

Preparation of the polyphenolic-rich extract (PESc) was performed as previously described with modifications [14]. Briefly, 300 g of powdered dried leaves was extracted by maceration with ethanol : water (70 : 30, *v*/*v*) under constant stirring at 25°C and filtered after 24 hours. This procedure was repeated twice, totaling three extractions. Extracts were then combined and centrifuged at 3500 rpm for 10 minutes at room temperature. The supernatant was concentrated in a rotary evaporator at 38°C to obtain the hydroethanolic extract (HESc). HESc was partitioned with chloroform (1 : 1 *v*/*v*; 3x), with subsequent partition of the aqueous phase with ethyl acetate (1: 1 *v*/*v*; 3x). The ethyl acetate fraction was concentrated under vacuum and lyophilized, yielding a polyphenol-enriched extract (PESc).

### 2.4. In Vivo Model of Alloxan-Induced Diabetes

To perform *in vivo* studies using the PESc, male Wistar Hannover rats (*Rattus norvegicus*) of 60 days were used. Rats were hosted in polypropylene boxes, in a maximum of 5 animals per box, maintained at 21 ± 2°C, 12 h light/dark cycles with food and water ad libitum. After adaptation to these conditions for a week, rats were randomly divided into three groups, and at day 7, all of them received a single ip injection of alloxan (150 mg/kg; Sigma Chem. Co., St. Louis, USA) after 12 hours of fast to induce diabetes [13, 15]. To prevent a fatal hypoglycemic state due to a fast release of insulin, the animals received 10% glucose in water for 12 h and food included an hour after alloxan injection. The three groups at day 7 were divided as follows: (i) ALX: rats who received alloxan at day 7 and then did not receive any other treatment than NaCl 0.9 g/L (1 mL/kg/d, *n* = 9); (ii) ALX-P: rats who received alloxan at day 7 and then 50 mg/kg/d PESc by gavage (*n* = 14) until the animals were euthanized; and (iii) ALX-PP: rats who received from day 0 50 mg/kg/day PESc by gavage (*n* = 14) and continued receiving the extract after the injection of alloxan. All the protocols used in this paper were approved by the institutional Ethical Committee on Animal Use (CEUA-UFMA), under ruling number 016/13. During the 14 days of treatment, the rats were weighted every day. Blood glucose levels were determined at days 1, 4, 7, 10, and 14 after overnight fasting, and periphery blood was collected by puncture of the caudal vein to determine glycemic levels using a blood glucose test strip (Accu-Chek Active, Roche Diagnostics, Mannheim, Germany).

### 2.5. Analysis of Lipid Profile and Triglyceride/Glucose Ratio

At day 14, the animals were overnight fasted and anesthetized and blood was collected from aorta puncture and used for triglyceride (TG) and total cholesterol (TC) determination. The serum levels of these lipids were determined by enzymatic commercial kits [16]. Insulin sensitivity was inferred by determining the triglyceride/glucose index (TyG) in accordance with the following calculation: TyG = Ln([TG(mg/dL)] × [glucose(mg/dL)]/2) [17].

### 2.6. Quantification of Total Phenols and Flavonoids

Total phenolic content was determined as described elsewhere [18]. Briefly, 100 *μ*L of PESc in 96% ethanol was mixed with 630 *μ*L of deionized water, 20 *μ*L of HCl 1 M, 150 *μ*L of 1% K_3_[Fe(CN)_6_], 50 *μ*L of 1% sodium dodecyl sulfate (SDS), and 50 *μ*L of 0.2% FeCl_3_·6H_2_O. Samples were vortexed and allowed to stand at room temperature for 30 minutes. The polyphenol content of PESc was determined by absorbance at 750 nm using a UV-VIS spectrophotometer (SP-2000 UV Spectrum); quantitation was performed using a gallic acid calibration curve. Results were expressed as gallic acid equivalents in grams per 100 g of dry extract (GAE/100 g).

In addition, flavonoid concentration was determined according to the previous method with modifications [19]. 200 *μ*L of 1 mg/mL samples in methanol was incubated with 5% aluminum chloride (1/1, *v*/*v*) and reached a final volume of 2 mL with methanol. The reaction mixture was incubated for 30 minutes at room temperature and protected from light. Flavonoids were determined spectrophotometrically at 425 nm in a UV-VIS spectrophotometer (SP-1105, Spectrum) and quantified using a calibration curve with quercetin. Results are expressed as quercetin equivalents per 100 g of dry extract (QE/100 g). Both procedures were repeated at least four times in separate experiments.

### 2.7. Isolation and Identification of Polyphenols by HPLC-MS/MS

Chemical characterization of polyphenolic compounds present in PESc was performed by HPLC-MS/MS analysis. Polyphenols were separated using a quaternary pump HPLC (Agilent 1260), and separation was performed in an analytical Phenomenex Luna C18 column (250 × 4.6 mm, 5 *μ*m). Before the run, extracts were filtered (0.45 *μ*M, Millipore) and dissolved in the same solvent used for extraction. The solvent system consisted of H_2_O/0.1% formic acid (solvent A) and acetonitrile/0.1% formic acid (solvent B) according to the following elution gradient at 5% B (0–1 min) and 5–30% B (1–30 min) and reequilibrated to initial conditions. The flow rate was 1 mL/min, and the column was maintained at 35°C [20]. From PESc, different fractions were collected and the solvent was evaporated and resuspended in methanol for MS/MS characterization. Mass spectrometry chemical characterization of polyphenols present in PESc was done by using a triple quadrupole ion trap QTRAP 4500 (AB Sciex, Framingham, MA). Samples were characterized in the negative mode with electrospray ionization (ESI) of the samples. Using purified standards, parameters for each compound were obtained by injecting samples at a flow rate of 10 *μ*L/min, a declustering potential (DP) of −70 eV, and a desolvation temperature of 350°C. For fragmentation analysis, the collision energy (CE) used was between −25 and −37 eV. HPLC-ESI-MS/MS of PESc was performed in the negative ion mode using the multiple reaction monitoring (MRM) scan mode. For LC-MS/MS experiments, the following parameters were used at the mass spectrometer DP of −100 V, CE of −25 to −37 eV, and a desolvation temperature of 550°C. The MRM transitions used were *m/z* 169/125 for gallic acid; *m/z* 317/271, *m/z* 317/151, and *m/z* 317/179, for myricetin; *m/z* 463/316, *m/z* 463/151, *m/z* 463/179, *m/z* 449/316, *m/z* 449/271, and *m/z* 449/179 for glycosylated derivatives of myricetin; and 301/151 and *m/z* 301/179 for quercetin [21, 22]. Analysis and processing of the obtained data were performed using the Analyst 1.6.1 software (Applied Biosystems, Framingham, MA).

### 2.8. Quantification of Main Polyphenols Identified in PESc

Using standards of gallic acid, myricetin, and quercetin, calibration curves by HPLC-UV were made. Standards and PESc separation and analysis were performed using a quaternary pump HPLC (Dionex UltiMate 3000) with an autosampler and a diode array detector. Detection of the different standards and the polyphenols present in PESc was done at 254 nm, 292 nm, 354 nm, and 375 nm while chromatographic conditions were the same for LC-MS/MS studies. For quantification purposes, gallic acid and myricetin calibration curves were done at 254 nm while that of quercetin was quantified at 375 nm [23, 24].

### 2.9. Analysis of Antioxidant Activity

#### 2.9.1. Scavenging of DPPH^•^

The antioxidant activity was evaluated by incubating 100 *μ*L of PESc with 1900 *μ*L of a methanolic solution of DPPH^•^ (25 *μ*g/mL) leading to a final extract concentration of 0.25–20 *μ*g/mL. The antioxidant activity was measured spectrophotometrically at 515 nm [25]. DPPH^•^ was prepared just before the beginning of experiment and kept in an amber bottle. The percentage of inhibition was calculated according to

(1) $$\begin{array}{c} Inhibition=\left\lceil \frac{A\;control–A\;sample}{A\;control}\right\rceil \times 100, \end{array}$$

where A is the absorbance. IC_50_ (effective concentration for 50% inhibition of preformed radical) was determined by nonlinear regression.

#### 2.9.2. Scavenging of ABTS^•+^

The ABTS solution was prepared in water and potassium persulfate and kept in the dark for 16 hours before testing for the complete oxidation of ABTS and the generation of the highly stable chromophore cation radical 2,2′-azino-bis(3-ethylbenzothiazoline-6-sulfonic acid) (ABTS^•+^) [26]. The ABTS^•+^ solution was diluted with absolute ethanol until the absorbance at 734 nm reached 0.7 ± 0.02. Readings were performed by reacting 1–20 *μ*g/mL of PESc with the ABTS^•+^ solution. All studies were performed at least in triplicate monitoring the decrease in absorbance for 10 min; results reported corresponded to the % of remaining chromophores compared to conditions in the absence of antioxidants. The IC_50_ values were determined as previously.

### 2.10. Analysis of Lipoxygenase Activity

Lipoxygenase (LOX) activity was measured spectrophotometrically at 234 nm [27]. The reaction was performed in 100 mM borate buffer pH 9 at 25°C in the presence of 100 *μ*M arachidonic acid (AA) prepared in 0.4% deoxycholate. The reaction was initiated with the addition of AA to the enzyme, and the increase in absorbance at 234 nm was monitored for 5 minutes under continuous stirring against a blank sample. Enzyme activity was determined from the initial linear slope. To analyze the effects of PESc on LOX, either the extract or the pure standards were added 2 minutes before enzyme activity was initiated. In all cases, specific LOX activity was determined by previously quantifying the amount of protein used in each experiment. Protein content was determined spectrophotometrically at 595 nm using the Bradford method, with BSA as standard [28]. The results shown were reported as the remaining specific LOX activity compared to the condition in the absence of extract or standard.

### 2.11. INS-1E *β* Cell Culture and Cell Proliferation Assay

INS-1E *β* cells were cultured in a humidified atmosphere (95%) containing 5% CO_2_ in complete RPMI 1640 medium (Sigma-Aldrich, Canada). These were supplemented with 1 mM sodium pyruvate, 50 mM 2-mercaptoethanol, 1 mM L-glutamine, 5% inactivated fetal calf serum (HyClone, Logan, UT), and 1 U/mL and 1 mg/mL of penicillin and streptomycin, respectively (Sigma-Aldrich, Canada). To determine cell proliferation, INS-1E *β* cells were seeded at 2 × 10^5^ cells/well density and incubated with PESc at increasing concentrations (1–1000 *μ*g/mL) for 48 hours at 37°C. After the incubation period, BrdU solution (10 *μ*M) was added for another 4 hours. DNA incorporation by BrdU was measured through colorimetric assay and carried out as per the manufacturer (Roche Diagnostics, Mannheim, Germany).

### 2.12. Glucose-Stimulated Insulin Secretion in INS-1E *β* Cells

INS-1E *β* cells were cultured as the abovementioned. To examine PESc effects on insulin secretion *in vitro*, a freshly diluted and filtered PESc solution was incubated at 100 and 1000 *μ*g/mL for 4 hours in a 96-well plate seeded with 2 × 10^5^ cells/well INS-1E *β* cells at 37°C. The medium was then washed twice with PBS and replaced by glucose-free Krebs-Ringer bicarbonate HEPES (KRBH) buffer (135 mM NaCl, 3.6 mM KCl, 5 mM NaHCO_3_, 1.5 mM CaCl_2,_ 0.5 mM NaH_2_PO_4_, 0.5 mM MgCl_2_, 1.5 mM CaCl_2_, 10 mM HEPES, and 0.1% BSA, pH 7.4) for 1 hour. Then, 2.8 mM glucose was added to KRBH for 30 min, washed with PBS, and incubated for 2 hours with 3.3 or 16.5 mM glucose KRBH. Finally, supernatants were collected and stored at −20°C until analysis. Insulin was measured with an ultrasensitive rat insulin ELISA kit and all procedures carried out as per the manufacturer (Crystal Chem Inc., USA).

### 2.13. Statistical Analysis

The results were expressed as mean ± standard error of the means with values of at least three independent experiments and analyzed with GraphPad Prism® (version 5.0). Statistical analyses were performed by Student's *t*-test or analysis of variance (ANOVA) followed by Tukey posttest. Differences were significant when *p* ≤ 0.05.

## 3. Results

### 3.1. PESc Reduction of Alloxan-Induced Hyperglycemia

**Figure 1:**
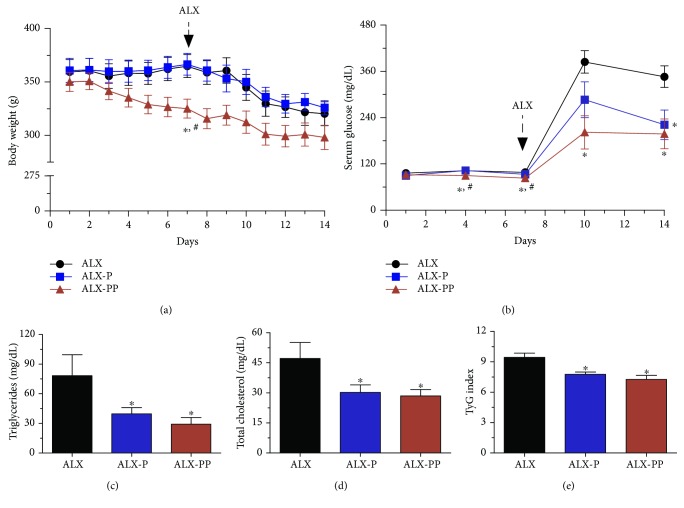
Effects of PESc in an *in vivo* diabetic model. Male Wistar rats (black squares, ALX, *n* = 9) were treated with PESc (50 mg/kg/d) from day 7 (blue squares, ALX-P, *n* = 14) or from day 0 (red triangles, ALX-PP, *n* = 14). At day 7, all animals received an injection of alloxan (150 mg/kg, ip) to induce diabetes as explained in the Materials and Methods section. During the experimental procedures, changes in body weight (a), serum blood glucose (b), triglycerides (c), and total cholesterol (d) levels were determined. In addition, the TyG index (e) was obtained from data in (c) and (d). Results shown correspond to the mean ± SEM; ^∗^*p* < 0.05 versus ALX and ^#^*p* < 0.05 versus ALX-P.

We evaluated the protective effects of PESc in an *in vivo* oxidative stress-induced diabetic rat model (Figure 1). Diabetic state was induced by the oxidative environment exerted by the ip injection of alloxan at day 7 [13]. When animals were weighted, rats who received PESc from day 0 (ALX-PP) showed body weight loss from 350.10 ± 7.48 g to 325 ± 7.69 g before alloxan injection at day 7 (Figure 1(a)). Neither ALX nor ALX-P groups showed a decrease in body weight during the first 7 days (Figure 1(a)). Importantly, glycemia was lower at days 4 and 7 in ALX-PP as compared to ALX and ALX-P, suggesting a hypoglycemic activity of the extract (Figure 1(b)). As expected, alloxan promoted body weight loss on all groups, though ALX-PP data suggest a lower slope of body weight decrease when compared to the other two groups (Figure 1(a)). Upon alloxan administration, fasting glucose levels measured in ALX rats at day 14 were 3.5-fold higher than those at day 7 (346.33 ± 27.89 mg/dL versus 98.22 ± 4.66 mg/dL, Figure 1(b)). For groups receiving PESc, fasting glucose levels verified at day 14 were strongly decreased in 37% for ALX-P and 43% for the ALX-PP group, as compared to the ALX group (221.64 ± 38.30 mg/dL for ALX-P and 197.92 ± 38.54 mg/dL for ALX-PP, Figure 1(b)).

### 3.2. PESc Prevents Insulin Resistance on Alloxan-Treated Rats

Administration of PESc lowered serum TG levels, reaching a maximum decrease of 63% on ALX-PP and 49% in ALX-P when compared to ALX (Figure 1(c)). Similar effects were observed for TC determinations which were reduced from 47.18 ± 7.97 mg/dL on ALX rats to 29.10 ± 3.09 mg/dL and 29.96 ± 3.53 mg/dL on ALX-P and ALX-PP, respectively (Figure 1(d)). Using fasting glucose and TC levels at day 14, we calculated the TyG index, which suggested decreased hepatic insulin resistance in ALX-P and ALX-PP, since TyG values were significantly reduced by 18% and 23%, respectively.

### 3.3. Total Antioxidant Capacity of PESc

**Figure 2:**
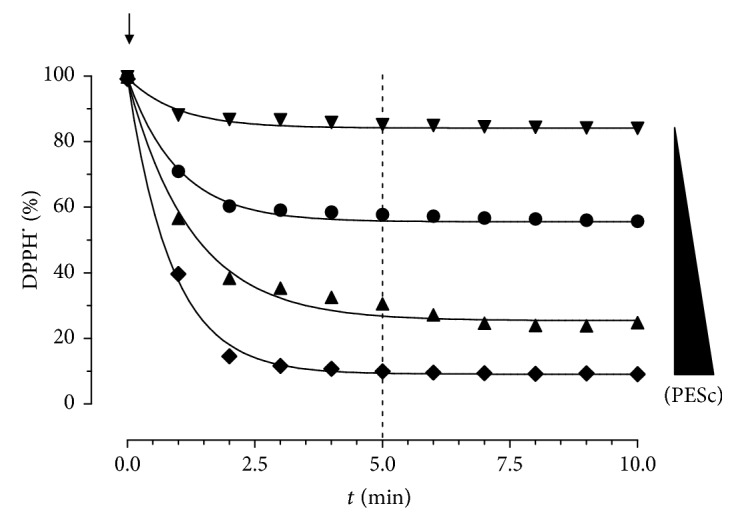
Kinetics of DPPH^.^ radical consumption after addition of polyphenol-rich extract (PESc). PESc (0.62–5 *μ*g/mL) was incubated with DPPH^.^ and the reaction monitored for 10 minutes to determine the maximum antioxidant activity. After 5 minutes, maximal effect was reached at all the concentrations tested as shown by the vertical dashed line.

Levels of polyphenols and flavonoids in PESc were determined spectrophotometrically, reaching values of 71.78 ± 8.57 GAE/100 g and 8.21 ± 0.42 QE/100 g, respectively. Due to the observed protection in an oxidative stress-mediated model of pancreatic damage, we next analyzed the total antioxidant potency of PESc by using the DPPH^•^ method (Figure 2). Since PESc reached the maximum scavenging effect before 5 minutes, it can be considered as having high antioxidant potency, with a concentration-dependent effect (Figure 2).

### 3.4. Identification of Polyphenols by HPLC-MS/MS

**Figure 3:**
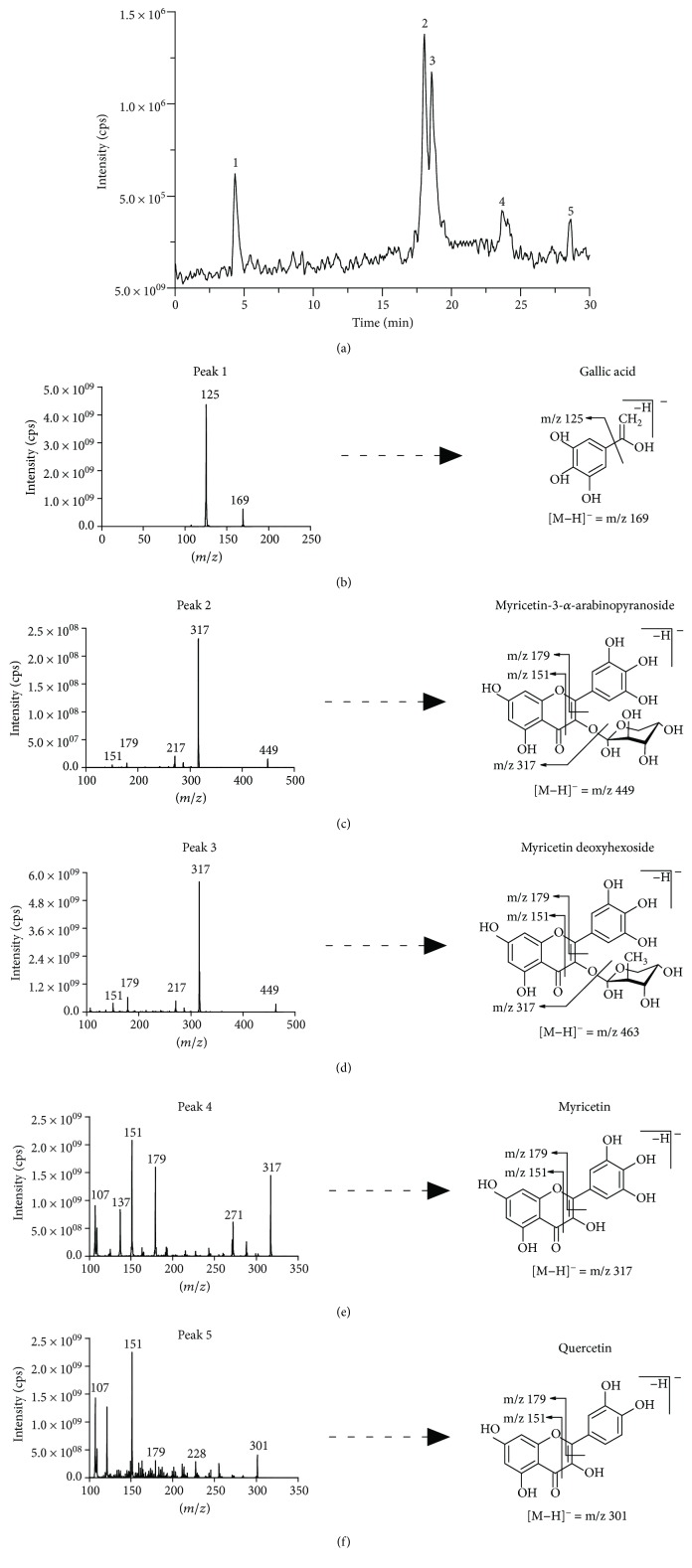
LC-MS/MS analysis of PESc polyphenol content. RP-HPLC-MS/MS chromatographic profile of PESc (a) was obtained. PESc was analyzed by MS/MS using the MRM method. Gallic acid was detected by using *m/z* 169/125, *m/z* 169/97, and *m/z* 169/79. For myricetin, MRM transitions corresponded to *m/z* 317/271, *m/z* 317/151, and *m/z* 317/179 while for glycosylated forms of myricetin *m/z* 449/316, *m/z* 449/271, and *m/z* 449/179. Finally, quercetin was followed by the *m/z* 301/151 and *m/z* 301/179. Chemical identification of the structure of the compounds present in PESc was analyzed by mass spectrometry. Representative structures and fragmentation patters of gallic acid (b); myricetin-3-*α*-arabinopyranoside (c), myricetin deoxyhexoside (d), myricetin (e), and quercetin (f) are shown. Data is representative of at least three independent experiments.

Once antioxidant capacity was verified, we moved to the chemical identification and characterization of compounds present in PESc which could be responsible for such activity (Figure 3).  Figure 3(a) shows the chromatographic elution profile of PESc with MS/MS detection. We were able to isolate, collect, and identify five peaks (named 1 to 5) whose structure and identity were obtained by mass spectrometry studies (Figures 3(b)–3(f)). MS/MS spectra of peak 1 showed the presence of the molecular negative ion ([M–H]^−^; *m/z* 169) which fragmented into an ion of *m/z* 125 (Figure 3(b)), characteristic of gallic acid [29]. The spectrum obtained was identical to the one obtained using a pure standard of gallic acid (not shown). Peaks 2 and 3, in accordance to their MS/MS spectra, were suggested to be myricetin derivatives (Figures 3(c) and 3(d)). Peak 2 exhibited a [M–H]^−^ of *m/z* 449 with a fragmentation pattern accordingly to myricetin-3-*α*-arabinopyranoside mainly formed by the presence of *m/z* 317, *m/z* 271, and *m/z* 179 ions (Figure 3(c)). The MS/MS spectrum presented in Figure 3(d) exhibited a [M–H]^−^ of *m/z* 463 which suggested the presence of myricetin deoxyhexoside whose aglycone has been detected from the fragment of *m/z* 317 [21]. Comparing with the available standard, peak 4 corresponded to myricetin due to its chromatographic retention time in addition to the MS/MS analysis (*m/z* 317 and fragments with *m/z* 179 and *m/z* 151; Figure 3(e)). The final compound characterized was quercetin, whose spectra presented a [M–H]^−^ of *m/z* 301 and fragments of *m/z* 179 and *m/z* 151 (Figure 3(f)). Finally, we quantified gallic acid, myricetin, and quercetin in PESc (Table 1). Myricetin was the most abundant flavonoid, accounting for nearly 20% of total PESc mass. Altogether, these data characterize the polyphenol content of PESc with abundancy of myricetin, gallic acid, and quercetin.

### 3.5. Antioxidant Activity of the Polyphenolic Compounds of PESc

**Figure 4:**
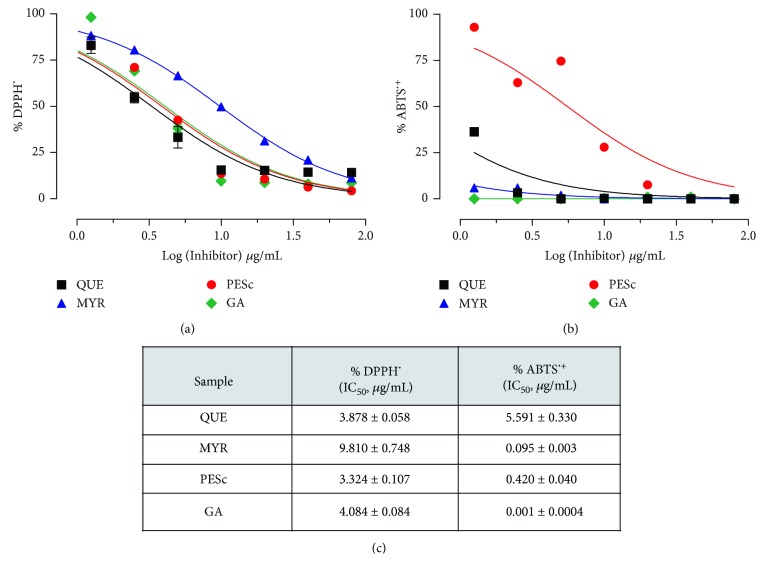
Antioxidant activity of PESc. The antioxidant capacity of PESc as well as the pure standards was determined by using DPPH (a) and ABTS (b) assays. In both cases, the concentration of extract or fractions leading to a decrease of 50% of initial activity (IC_50_) was determined (c). Data shown are representative of at least three independent experiments, performed by triplicate for each condition, and expressed as the mean ± SEM.

Since PESc is rich in myricetin and other flavonoids, we next analyzed the antioxidant activity of PESc in comparison to gallic acid, myricetin, and quercetin standards by using DPPH^•^ (Figure 4(a)) and ABTS^•+^ (Figure 4(b)) assays. As previously determined, antioxidant reactions were followed for up to five minutes. For the DPPH^•^ method, PESc showed an IC_50_ similar to that measured for quercetin and gallic acid and two times lower than that for myricetin (Figure 4(c)). Using the ABTS method (Figure 4(b)), PESc presented an antioxidant activity at least ten times lower than pure standards (Figure 4(c)).

### 3.6. Inhibition of 12/15 Lipoxygenase Activity

**Figure 5:**
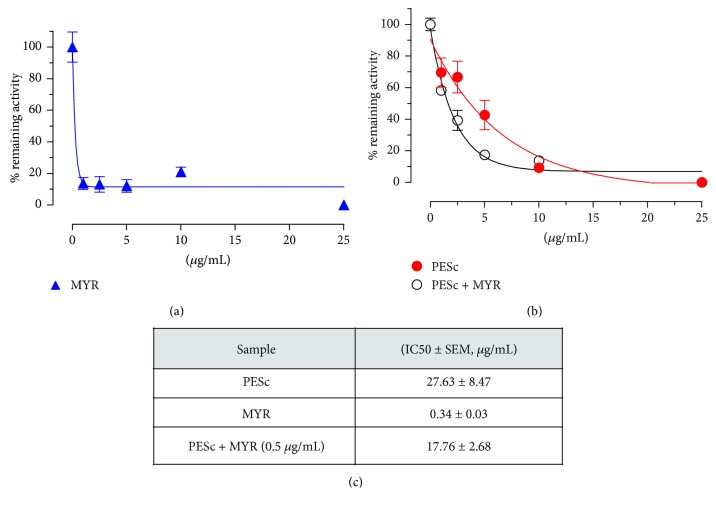
Inhibition of LOX by PESc. Lipoxygenase activity was followed by formation of conjugated dienes at 234 nm (*ε* = 2.5 × 10^−4^ M^−1^·cm^−1^). Activity was determined from the initial linear slope of changes in absorbance. Enzyme protein concentration was determined and specific activity in the absence or presence of the extract or the purified fractions reported. Results are expressed as the remaining initial activity in the presence of the different inhibitors' concentrations. (a) Dose-dependent action of myricetin (MYR) on LOX activity. (b) LOX activity was determined at different PESc concentrations in the absence (closed squares) or presence (open circles) of MYR, to detect if an additive or cooperative action was observed. (c) The obtained IC_50_ values in each condition are reported. In all cases, data are presented as mean ± SD of three independent experiments at least in triplicate for each condition.

LOX is a biologically relevant enzyme widely present in plants and mammals that catalyzes the oxidation of unsaturated fatty acids, that is, AA and linoleic acid. By following changes in absorbance at 234 nm, we analyzed the capacity of either PESc and/or the standards to inhibit 12/15 LOX activity (Figure 5). Both myricetin (Figure 5(a)) and PESc (Figure 5(b)) were able to inhibit 12/15 LOX activity in a concentration-dependent manner in a process that was enhanced by the supplementation of PESc with myricetin (Figures 5(b) and 5(c)). Neither gallic acid nor quercetin inhibited 12/15 LOX activity (data not shown).

### 3.7. Cell Proliferation and Insulin Secretion

**Figure 6:**
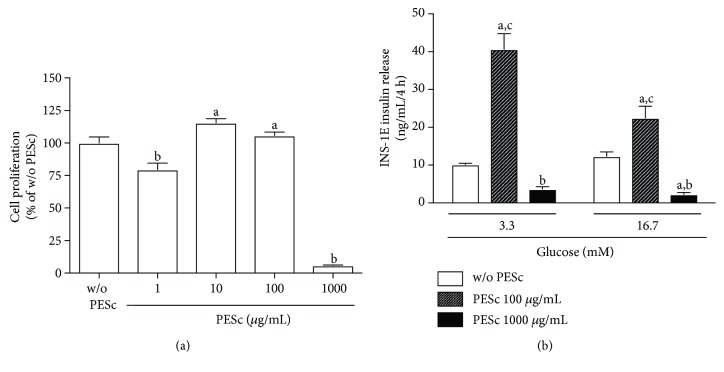
Cell proliferation and insulin release induced by PESc. Cell proliferation was determined by BrdU incorporation to DNA, whereas insulin levels were determined by commercial ELISA kit—both described in the Materials and Methods section. (a) Cell proliferation after 48 hours of incubation with or w/o PESc. Results expressed as relative to w/o PESc condition. (A) *p* < 0.05 versus w/o PESc and (B) *p* < 0.05 versus all. *n* = 9 per group. (b) Glucose-stimulated insulin secretion (GSIS) in INS-1E insulinoma cells incubated with 100 or 1000 *μ*g/mL PESc for 4 hours at 37°C. (A) *p* < 0.05 versus w/o PESc; (B) *p* < 0.05 versus PESc 100 *μ*g/mL; (C) *p* < 0.05 versus PESc 1000 *μ*g/mL. *n* = 8 per group. All data were analyzed by one-way ANOVA followed by Tukey posttest and presented as mean ± SEM.

Due to the beneficial effects of PESc on alloxan-induced diabetes and its potent antioxidant activity, we determined whether this extract would have any effect on *in vitro* INS-1E *β* cell culture proliferation and insulin secretion. After 48 hours of incubation, PESc did not compromise cell proliferation up to 100 *μ*g/mL, as measured by BrdU incorporation to DNA (Figure 6(a)). At 1000 *μ*g/mL, PESc incubation abolished cell proliferation probably due to an extremely high concentration. Importantly, acute incubation of 100 *μ*g/mL PESc induced a 4-fold increase in basal insulin secretion and a 2-fold increase in 16.7 mM glucose-stimulated insulin secretion (Figure 6(b)). Altogether, these results provide evidence that the positive effects of PESc seen *in vivo* are not only due to its antioxidant capacity, but may also be a consequence of increased insulin secretion by *β* cells.

## 4. Discussion

The present study strengthens the chemical and pharmacological knowledge of the phytochemical composition of *S. cumini* leaf as well as its applicability in complementary management of cardiometabolic disorders. Data herein described show that PESc, a novel polyphenol-rich extract prepared from *S. cumini* leaf, presented a protective effect on a model of oxidative stress-induced diabetes. These effects were possibly due to PESc potent antioxidant capacity, demonstrated by a prominent ability to scavenge DPPH^•^ and more importantly to inhibit the biologically relevant 12/15 LOX enzyme. Chemical characterization evidenced abundance of gallic acid, quercetin, and myricetin derivatives. Moreover, PESc induced insulin secretion by INS-1E *β* cells, without affecting cell proliferation at potentially therapeutic concentrations.

Alloxan produces a diabetic state through the accumulation of reactive oxygen species on pancreatic *β* cells causing detrimental effects since *β* cells have incipient antioxidant defense [13]. Upon alloxan injection, all rats presented a marked increase in fasting serum glucose levels consequent to the pancreatic oxidative damage. However, rats from ALX-P and ALX-PP groups had significantly lower increases than those from the ALX group, depicting the ability of PESc to attenuate alloxan-induced diabetes regardless of when PESc was administered. Accordingly, PESc administration reduced fasting serum levels of TG and TC in both ALX-P and ALX-PP groups, an effect further assessed by the improved hepatic insulin sensitivity inferred from TyG index calculation. These findings are in line with our previous report of the dual effect of a hydroethanolic extract from *S. cumini* leaf on insulin secretion and peripheral insulin sensitivity of MSG-obese rats [12]. Studies carried out with *S. cumini* seed extract in HepG2 cells [14] and liver from streptozotocin-induced diabetic rats [30] showed hypolipidemic effects, which were attributed to increased PPAR*γ* activity and expression. Importantly, myricetin, a flavonoid widely distributed through *S. cumini* tree, has been shown to improve insulin sensitivity [31] and promote hepatic lipid oxidation by increasing PPAR*α* expression in the liver [32]. Nevertheless, the protective effect of PESc could also come from a direct improvement of cellular redox balance, similar to the islet-regenerative potential shown for a purified fraction of *S. cumini* seeds in streptozotocin-induced diabetic mice [33]. Therefore, here we showed that acute PESc administration before or after diabetes induction was sufficient to attenuate diabetic state.

To investigate whether PESc *in vivo* effects were due to its antioxidant activities, we first characterized PESc polyphenolic content. PESc presented a polyphenol content 3-fold higher than that of HESc [12] and virtually even higher than those reported for different extracts prepared from *S. cumini* seeds [34]. In fact, values reported by Arun et al. [34] may be overestimated, since they were obtained using the Folin-Ciocalteu method, which also quantifies nonphenolic compounds such as aromatic amino acids, sugars, ascorbic acid, and organic acids, the reason why it is no longer advised for total phenol quantification [18]. LC-MS/MS studies of PESc allowed us to identify and quantitate the compounds gallic acid, myricetin, and quercetin, showing that approximately 30% of PESc total mass was made of these three compounds. As expected for an RP chromatography, gallic acid eluted first whereas quercetin was the last compound to elute from the column, explained by different polarities between compounds [35].

Antioxidant capacity of *S. cumini* leaf has been formerly shown for hydroethanolic [12] and aqueous [36] extracts, whereas PESc apparently present higher capacity. Both quercetin and gallic acid showed similar scavenging capacity of DPPH^•^ compared to the extract, while myricetin presented higher IC_50_. The low IC_50_ found for PESc on DPPH^•^ assay corroborates the novelty of PESc, since there is no data on literature describing such results for a *S. cumini* extract. In fact, PESc scavenging capacity is at least three times higher than those described in different reports [37, 38]. For ABTS^•+^ assay, the standards showed a stronger antioxidant activity when compared to PESc. Despite this, as far as we know, PESc showed the lowest IC_50_ against ABTS^•+^ for a *S. cumini* extract described thus far [39, 40]. ABTS^•+^ is reactive through both hydrophilic and hydrophobic radicals while DPPH^•^ assay is performed in organic solvents where hydrophobic radicals reduce the radical [41]. As expressed before for their chromatographic behavior, polarity of the tested polyphenols is different, supporting the distinct lipophilicity and reactivity of the compounds present in PESc [36]. In addition, it has been reported that flavonoids exert different antioxidant properties due to the localization of hydroxyl groups at C-3 and C-3′ on flavonoid B ring [42]. Steric impediments were also suggested to impact the reactivity of gallic acid, quercetin, and myricetin with different oxidant models [42], which can also explain the different activities seen against DPPH^•^ and ABTS^•+^.

Since our antioxidant capacity studies used nonbiologically relevant oxidants, we next decided to assess PESc action on the enzymatic lipid-derived oxidation of AA by 12/15 LOX, whose signaling pathway encompasses a variety of cellular pro- and anti-inflammatory mediators, such as hydroperoxyeicosatetraenoic or hydroxyeicosatetraenoic acids, leukotrienes, and lipoxins [43]. Moreover, the catalytic mechanism of AA oxidation by LOX involves the formation of a lipid-derived radical, which can be sequestered or reduced by the biologically active polyphenols present in PESc. PESc could concentration-dependently inhibit LOX activity, an effect further enhanced upon addition of 0.5 *μ*g/mL myricetin, leading to a 35% reduction in IC_50_. A similar additive effect for quercetin supplementation was obtained only at concentrations 20-fold higher than that used for myricetin (data not shown). Moreover, gallic acid was unable to exert inhibition of enzymatic activity (data not shown). Thus, PESc inhibition of 12/15 LOX improves our understanding on the mechanisms of action of both extract and its constituent polyphenolic compounds.

Given the beneficial effects of PESc on an oxidative stress-induced diabetes model, coupled with its antioxidant activities *in vitro*, we sought to further understand the extract's effect on *β* cell culture. Importantly, PESc did not interfere with cell proliferation up to 100 *μ*g/mL, while promoting a potent insulinagogue effect, at both basal and glucose-stimulated conditions. These effects support the restoration of islet architecture, as well as the glucose-insulin axis seen on ALX-P and most evidently on ALX-PP animals, comparable to effects described for *S. cumini* seed extract on streptozotocin-induced diabetic mice [33]. In addition, it is known that 12/15 LOX is highly relevant in *β* cell insulin secretion, both at physiologic [44] and diabetic conditions [45]. Thus, by inhibiting 12/15 LOX, PESc might improve insulin secretion directly on *β* cells, even though other mechanisms cannot be ruled out. Notwithstanding, 12/15 LOX is also relevant in a broader setting of cardiovascular disease, for instance by promoting thrombus formation *in vivo* [46], which enlarges PESc potentiality as an interesting new tool to treat and prevent thromboembolic outcomes associated to metabolic disorders.

## 5. Conclusions

In conclusion, our study showed the *in vitro* and *in vivo* antioxidant activities of PESc obtained from *S. cumini* leaf in addition to the chemical characterization and identification of its main polyphenolic compounds, which could be responsible for the observed actions. Our findings support that myricetin, quercetin, and gallic acid compose a phytocomplex, with poorly understood synergistic mechanisms of action. Nevertheless, the strong attenuation of oxidative stress-derived metabolic outcomes observed *in vivo* using such a low dose of PESc definitely reinforces the potential use of this novel polyphenol-enriched extract from *S. cumini* leaf as a source of antidiabetic natural products.

## Figures and Tables

**Table 1:** Quantification of main polyphenols identified in PESc.

Compounds	Structure	*μ*g/mg of PESc
Gallic acid	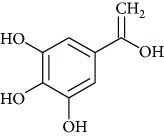	11.15 ± 0.90
Myricetin	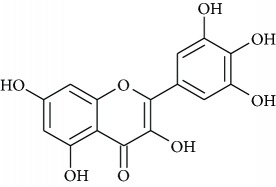	192.70 ± 16.50
Quercetin	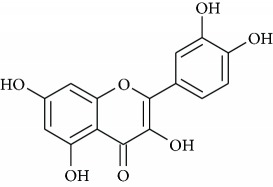	4.72 ± 0.06

Gallic acid (AUC_254nm_ = 624.40 [GA] + 17.25), myricetin (AUC_254nm_ = 132.90 [MYR] + 26.34), and quercetin (AUC_375nm_ = 1110.10 [QUER] − 36.42) were quantified by HPLC-UV. The retention times for those peaks used for quantitation purposes for each compound were previously identified by MS/MS as shown in Figure 3. Results are reported per mg of PESc and correspond to the mean ± SD from three different batches.

## Abbreviations

DPPH^•^: 2,2-Diphenyl-1-picrylhydrazyl-1
ABTS^•+^: 2,2′-Azinobis-(3-ethylbenzthiazoline sulfonic acid-6)
PESc: Polyphenolic-rich extract
HESc: Hydroethanolic extract
MRM: Multiple reaction monitoring
IC_50_: Effective concentration for 50% inhibition
LOX: Lipoxygenase
AA: Arachidonic acid
TG: Triglycerides
TC: Total cholesterol
TyG: Triglyceride/glucose index
BrdU: 5-Bromo-2′-deoxyuridine.
