# Corrigendum to “Protective Effects of a Polyphenol-Rich Extract from *Syzygium cumini* (L.) Skeels Leaf on Oxidative Stress-Induced Diabetic Rats”

**DOI:** 10.1155/2019/5785798

**Published:** 2019-01-09

**Authors:** Vinicyus Teles Chagas, Rafaella Moraes Rego de Sousa Coelho, Renato Simões Gaspar, Samira Abdalla da Silva, Mauricio Mastrogiovanni, Cáritas de Jesus Mendonça, Maria Nilce de Sousa Ribeiro, Antonio Marcus de Andrade Paes, Andres Trostchansky

**Affiliations:** ^1^Department of Physiological Sciences, Federal University of Maranhão, 65080805 São Luís, MA, Brazil; ^2^Facultad de Medicina, Universidad de la Republica (UdelaR), Montevideo, Uruguay; ^3^Department of Chemistry, Federal University of Maranhão, 65080805 São Luís, MA, Brazil; ^4^Department of Pharmacy, Federal University of Maranhão, 65080805 São Luís, MA, Brazil

In the article titled “Protective Effects of a Polyphenol-Rich Extract from *Syzygium cumini* (L.) Skeels Leaf on Oxidative Stress-Induced Diabetic Rats” [1], the second affiliation should have been written as “Facultad de Medicina, Universidad de la Republica (UdelaR), Montevideo, Uruguay.” Also, the name of the seventh author was given incorrectly as Maria Nilce de Souza Ribeiro. The author's name should have been written as Maria Nilce de Sousa Ribeiro. The revised authors' list and affiliations are shown above.
